# 

**Published:** 1856-02

**Authors:** 


### CONTENTS.

ORIGINAL COMMUNICATIONS— PAGE.

On the Climate of Lake Superior. By Charles Whittlesey, of Eagle River, Ill.,.......... 55
Cases in Practice. By Benj. Woodward, M.D., Sharon, Ill.......... 59
Iodine, a Remedy in Rattlesnake Bites,.......... 61
Cases of Puerperal Convulsions. By J. F. Beckner, M.D., of Blish Mills, Missouri,.......... 62
Remittent Insanity Cured by Quinine. By Dr. H. W. Kreider,.......... 64

SELECTIONS—

On the Use of the Tinct. Ferr. Chlorid. in Uterine Hemorrhages. By Dr. Frederick Schreier, Physician in Hamburgh,.......... 69
On Infantile Paralysis. By Wm. Adams,.......... 72
Treatment of Sciatica. By Peyton Blakiston, F.R.S.,.......... 75

BOOK NOTICES,.......... 81
EDITORIAL,.......... 87

